# Hidden colon adenocarcinoma diagnosed from mouth metastasis: case report and literature review

**DOI:** 10.1186/s12957-023-02978-y

**Published:** 2023-03-10

**Authors:** Maria Leticia de Almeida Lança, Yasmin Rodarte Carvalho, Janete Dias Almeida, Estela Kaminagakura

**Affiliations:** Department of Bioscience and Oral Diagnosis, Institute of Science and Technology, São Paulo State University (UNESP), Avenue: Engenheiro Francisco José Longo, 777, São José dos Campos, 1245-000 Brazil

**Keywords:** Adenocarcinoma, Maxilla, Neoplasm metastasis

## Abstract

**Background:**

We report an unusual case of metastatic colon adenocarcinoma to the maxilla as an initial clinical sign of the disease, this being the second case reported in the palate. In addition, we show an extensive review of the literature, with clinical cases of adenocarcinoma with metastasis to the mouth.

**Case presentation:**

An 80-year-old man complained of “swelling on the palate” with a 3-week evolution time. He reported suffering from constipation and high blood pressure. The intraoral examination revealed a pedunculated, red, and painless nodule on the maxillary gingiva. Under the diagnostic hypotheses of squamous cell carcinoma and malignant neoplasm of the salivary gland, an incisional biopsy was performed. Microscopically, the columnar epithelium was observed forming papillary areas, neoplastic cells with prominent nucleoli, hyperchromatic nuclei, atypical mitotic figures, and mucous cells, being positive for CK 20, suggesting the provisional diagnosis of metastatic adenocarcinoma, probably of gastrointestinal origin. The patient was submitted to endoscopy and colonoscopy exams, and a lesion in the sigmoid region of the colon was observed. After a colon biopsy, a moderately differentiated adenocarcinoma was confirmed, establishing the final diagnosis of metastatic neoplasia of colon adenocarcinoma to the oral lesion. The literature review revealed 45 clinical cases of colon adenocarcinoma with metastasis to the oral cavity. To the best of our knowledge, it is the second case on the palate.

**Conclusions:**

Colon adenocarcinoma with metastasis to the oral cavity is rare but should be included in the differential diagnosis of neoplasms of the oral cavity, even when there are no known primary tumors in some cases, and this may be the first indication of the presence of a tumor.

## Background

Metastatic tumors in the oral region are uncommon, comprising less than 1% of all malignancies [1]. Patients between the fifth and seventh decade of life are among those that most present metastases in the oral region; however, there is not a major difference between the sexes [2]. In the jaws, the mandible is the region most affected, while in the soft tissues, the inserted gum is the most frequently involved site [3, 4]. However, the frequency of the site of oral metastasis changes depending on the primary lesion location [2].

All types of malignant tumors can metastasize to the oral cavity, but the main tumors that present oral metastases are lung, kidney, liver, and prostate for men and breast, female genitals, and kidneys for women [3]. Colon cancer is the fourth most frequent cancer and second cancer concerning mortality worldwide [5]. Most colorectal neoplasias metastasize to the local lymph nodes, liver, and lungs [6].

Oral metastases are rare and relevant clinical studies are scarce, which makes diagnosis and treatment difficult [1, 7]. The prognosis for these patients is extremely poor, and the majority die within 9 months after the diagnosis [8]. The present case report highlights an unusual case of colon adenocarcinoma that metastasized to the maxilla, which is an early clinical sign of this disease. We also performed an extensive literature review encompassing clinical case reports of colorectal adenocarcinoma with metastasis to the mouth.

## Case presentation

**Fig. 1:**
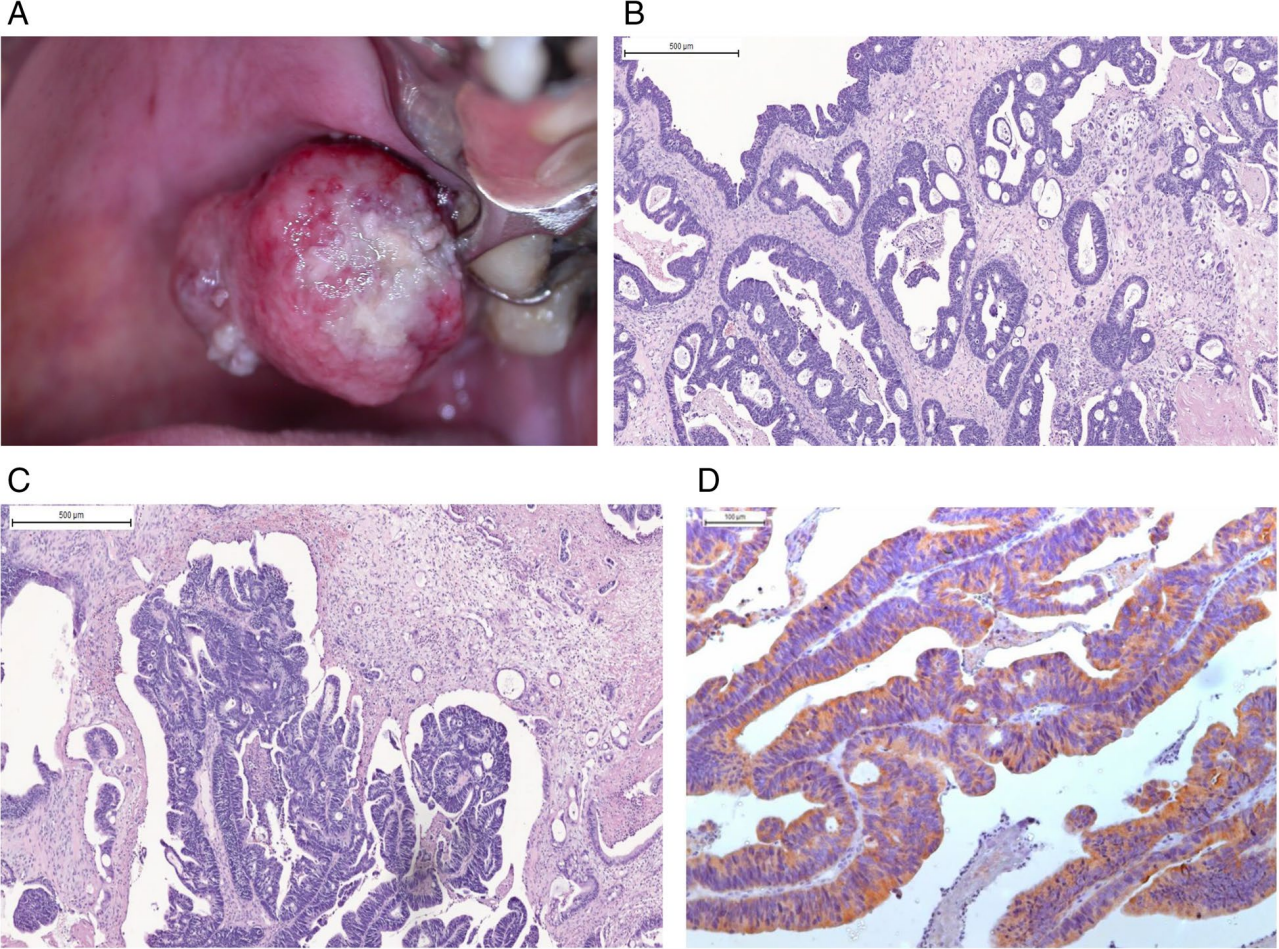
Clinical and histological findings. **A** Initial clinical photograph exhibiting a nodular lesion on the maxillary gingiva. **B** Microphotography revealed neoplastic cells with prominent nucleoli, hyperchromatic nuclei, mucous cells, and cystic formation. **C** Histopathological features showing columnar epithelium forming papilliferous areas and mucous cells. Hematoxylin and eosin stain. **D** Neoplastic cells present positivity for CK20 in the cytoplasm

An 80-year-old male presented with the principal complaint of “swelling on the palate” that appeared 3 weeks prior to examination. The patient’s significant medical history included constipation and hypertension. The patient was currently taking daily antihypertensive medication. He denied having any habits or addictions. In an intraoral examination, a reddish painless pedunculated nodule was observed with ulcerated areas located on the palate, between the left upper first and second molars, measuring 2 cm at its largest diameter (Fig. 1a). Radiographic examination showed no changes, with the underlying bone intact (Fig. 1b). The clinical diagnostic hypotheses of squamous cell carcinoma, malignant neoplasm of the salivary gland, and pyogenic granuloma were established. An incisional biopsy was performed, and histopathological analysis revealed columnar epithelium forming papilliferous areas, mucous cells, and cystic-like formation. The immunohistochemical analysis was positive for CK20. Neoplastic cells exhibited prominent nucleoli, hyperchromatic nuclei, and some atypical mitotic figures (Fig. 1c and d).

**Fig. 2:**
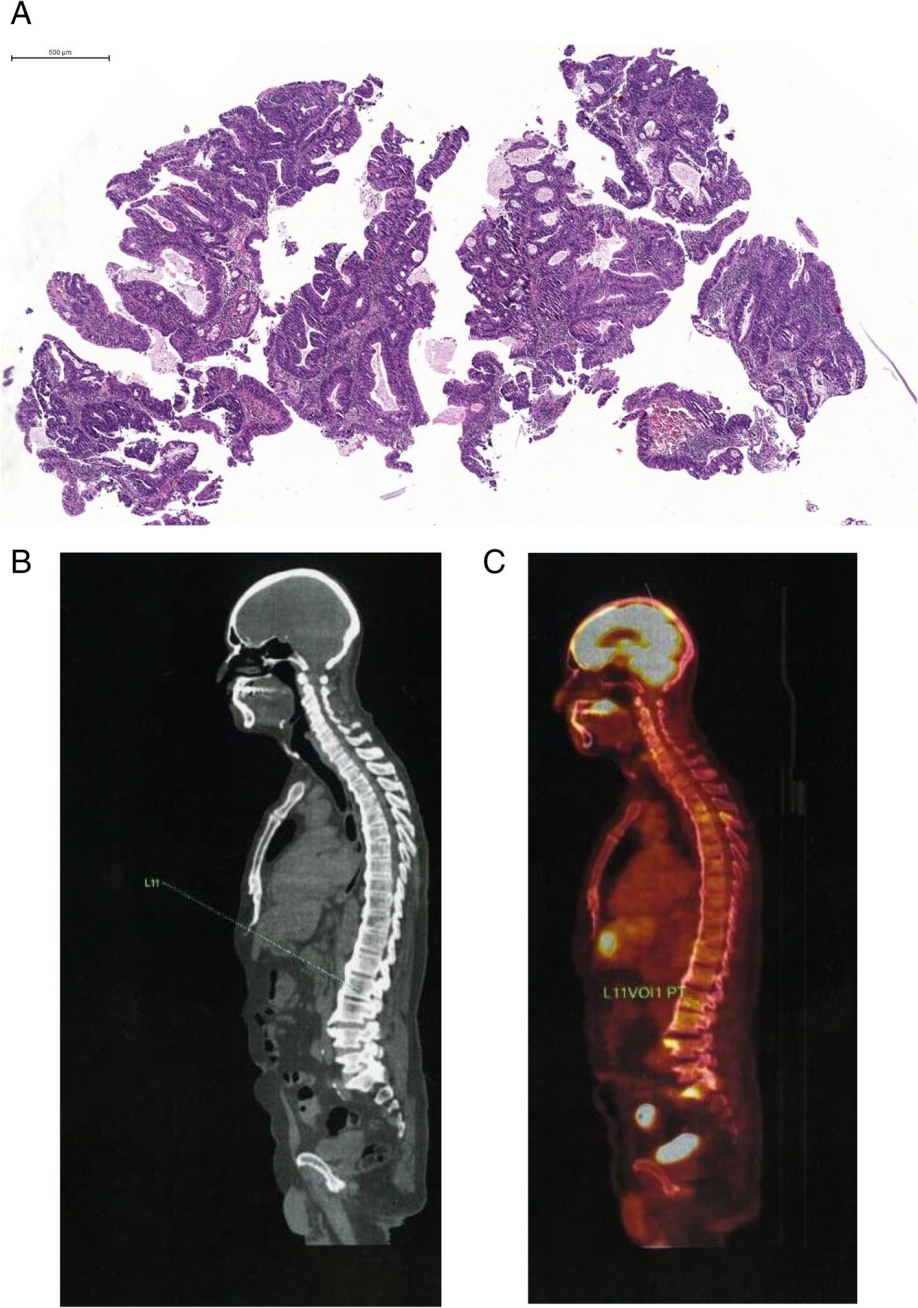
**A** Histopathological features of primary moderately differentiated adenocarcinoma from the colon (hematoxylin and eosin stain). **B**, **C** Positron emission tomography–computed tomography: uptake in the encephalon; hard palate, liver, lumbar 1 vertebral body, and sigmoid region

The histopathological features suggested metastatic adenocarcinoma probably of gastrointestinal origin. The patient was sent to the clinician for investigation concerning the origin of the primary tumor. Endoscopy and colonoscopy were performed, which showed a scar in the esophagus, chronic gastritis, and a lesion in the sigmoid region of the colon. After a colon biopsy, a moderately differentiated adenocarcinoma was confirmed (Fig. 2a). A positron emission tomography—computed tomography (PET-CT) showed increased uptake of F18-FDG in the brain, hard palate, liver, lumbar 1 vertebral body, and sigmoid region (Fig. 2b and c). Therefore, a final histological diagnosis confirmed the metastatic adenocarcinoma of the colon. The patient underwent 8 cycles of chemotherapy with 5-fluorouracil and 10 mg/mL calcium folinate. Unfortunately, he died 3 months later.

## Discussion and conclusions

We conducted an extensive literature review in search of articles on clinical cases of colorectal adenocarcinoma metastasis in the mouth. The search was performed on PubMed and in the references of selected articles, from inception through July 2022, with no language or publication date restrictions, and the strategy was limited to humans. Forty-one articles published between 1936 and 2020 were selected, reporting 45 clinical cases (Table 1), including the present case [1, 6–44]. It was observed that patients were between the seventh (33.33%) and eighth (31.11%) decades of life, and the age ranged from 33 to 80 years. There was a predominance of males, with 26 (57.78%) men and 19 (42.22%) women. In 10 (22.22%) patients, the diagnosis of metastasis was made before finding the primary tumor.

**Table 1:** Clinical information of colorectal adenocarcinoma metastasis in the mouth

Author(s)	Year	Age	Sex	Site of metastasis	Initial diagnosis	Follow-up	OS^a^ in months
[9] Humphrey, 1936	1936	33	Male	Maxillary gingiva	Primary	Died	0,43
[10] Bruce, 1954	1954	71	Female	Mandible	Primary	Died	6
[11] Meyer, 1958	1958	64	Female	Mandible	Primary	Died	6
[12] Clausen, 1963	2009	60	Male	Mandibular gingiva	Primary	Died	4
[13] Straith, 1967	1967	60	Female	Mandible	Metastasis	Died	2
[14] Levy, 1974	1974	80	Female	Mandibular gingiva	Primary	Died	1
[15] Moffat, 1976	1976	56	Male	Mandibular gingiva	Primary	Died	NR
[16] Rentschler, 1982	1982	73	Male	Mandibular gingiva	Primary	Alive	-
[17] Giles, 1982	1982	55	Female	Condyle	Primary	Died	7
[18] Delfino, 1982	1982	65	Male	Mandible	Primary	Died	Several weeks
[19] Rusthoven, 1984	1984	45	Female	Mandibular gingiva	Primary	Died	2,5
		65	Male	Maxillary gingiva	Primary	Died	5
[20] Naylor, 1989	1989	65	Female	Mandibular gingiva	Primary	NR	NR
[21] Nitzan, 1990	1990	75	Female	Mandible	Metastasis	NR	NR
[22] Babu, 1996	1996	75	Male	Mandible	Metastasis	Alive	-
[23] Bentley, 1997	1997	70	Female	Zygoma	Primary	NR	NR
[24] Cantero, 1998	1998	79	Female	Mandibular gingiva	Metastasis	NR	NR
[25] Mason, 2005	2005	73	Male	Condyle	Metastasis	Died	NR
[26] Mojica-Manosa, 2006	2006	66	Male	Mandible	Primary	Died	2^b^
[27] Spinelli, 2006	2006	72	Male	Palate	Primary	NR	NR
[28] Álvarez-Álvarez, 2006	2006	62	Male	Mandibular gingiva	Primary	Died	9
[29] Kawamura, 2008	2008	51	Female	Maxillary gingiva	Primary	Died	2
[30] Iida, 2009	2009	55	Male	Mandibular gingiva	Primary	Died	11
[31] Bell, 2009	2009	58	Male	Base of the tongue	Metastasis	Alive	-
		58	Male	Base of the tongue	Metastasis	Alive	-
[32] Favia, 2010	2010	66	Female	Mandible	Primary	NR	NR
		35	Female	Mandible	Primary	NR	NR
[8] Soares, 2011	2011	42	Male	Mandibular gingiva	Metastasis	Alive	-
[6] Singh, 2011	2011	42	Female	Floor of the mouth	Primary	Died	NR
[1] Amin, 2011	2011	75	Male	Mandible	Primary	NR	NR
[33] Murugaraj, 2012	2012	70	Male	Mandibular gingiva	Metastasis	Died	9
[34] Lagha, 2012	2012	46	Male	Mandibular gingiva	Primary	Died	4
[35] Yang, 2014	2014	74	Female	Mandible	Primary	Died	3
[36] Miyake, 2015	2015	60	Female	Mandibular gingiva	Primary	NR	NR
[37] Baranović, 2015	2015	78	Male	Maxillary gingiva	Primary	Died	4
[38] Watanabe, 2016	2016	64	Male	Mandibular gingiva	Primary	Died	2^b^
[39] McGoldrick, 2016	2016	58	Male	Mandible	Primary	Alive	-
[7] Ren, 2017	2017	60	Male	Mandibular gingiva	Primary	NR	NR
[40] Romanet, 2018	2018	62	Male	Mandibular symphysis	Primary	Died	15
[41] Di Stasio, 2018	2018	74	Male	Maxillary gingiva	Primary	Died	6
[42] Pelissari, 2018	2018	64	Female	Mandibular gingiva	Primary	Died	6
[43] Dalirsani, 2020	2020	69	Female	Maxillary gingiva	Primary	Died	6
[44] Samlali, 2020	2020	79	Female	Mandible	Primary	Died	4
[45] Neumann, 2020	2020	59	Male	Maxillary gingiva	Primary	Died	10
Present case	2022	80	Male	Palate	Metastasis	Died	0,75

*NR* not reported

^a^Overall survival

^b^After treatment

Malignant tumors with oral metastases are uncommon [46]. In our patient, it appeared as an asymptomatic, reddish mass with ulcerated areas located on the upper maxillary gingiva, suggesting a differential diagnosis of squamous cell carcinoma, salivary gland neoplasm, or pyogenic granuloma. In the gingiva, the lesion can be similar to a hyperplastic or reactive lesion, such as pyogenic granuloma, peripheral giant granuloma, or fibrous epulis; in other oral soft tissues, it appears as a submucosal nodule, and in a few cases as ulceration [8, 43]. Metastases do not necessarily have a malignant clinical appearance, which can lead to a misdiagnosis and delay their treatment [8, 43]. However, cases of oral adenocarcinoma metastases had a median survival of 6 months after diagnosis. Our patient presented a fast progression, with death in 3 weeks.

What makes this case report unique is the presence of metastasis to the maxilla. There are eight cases reported in the English literature of adenocarcinoma metastasis to this location [9, 18, 26, 28, 37, 41, 43, 45]. Colon carcinomas usually metastasize to regional lymph nodes, liver, peritoneum, lungs, or ovaries, rarely in supraclavicular organs [47]. Although poorly understood, a possible mechanism that leads to metastasis to the mandibles is the Batson plexus [48]. There is free communication between the venous systems of the neck, thorax, abdomen, and pelvis with the vertebral venous plexus without valves that extends from the base of the skull to the coccyx. An increase in pressure in the abdomen can create an upward flow through the vertebral venous plexus and thus metastatic cells can reach the maxilla and mandible [48, 49]. However, it is not a simple mechanism due to the difference in disseminated metastases between the mandible bones and the oral mucosa, even though they share the same blood supply [3].

Our patient presented the lesion located on the maxillary gingiva. The gingiva is a site with chronic inflammation that favors circulating metastatic tumor cells [4]. Chronic inflammation is related to several stages of tumor formation, such as cell transformation, promotion, survival, proliferation, invasion, angiogenesis, and metastasis [50]. In this literature review, the most common site of oral metastases was the lower gingiva, with 18 cases (40.00%), followed by the mandible with 13 cases (28.88%). Bone metastasis of the mandible is found more frequently due to the existence of bone marrow in these regions, but to a lesser extent in older people [3, 51].

Metastases in the oral cavity can show rapid progression, pain, bleeding, or paresthesia [3, 52]. Furthermore, the histological examination, accompanied by other diagnostic approaches is important to establish the correct diagnosis as fast as possible [37]. The histological differential diagnosis can be made with sinonasal intestinal-type adenocarcinoma, which is morphologically similar to intestinal primary adenocarcinoma. Both can be indistinguishable on histological analysis, but can be differentiated with immunohistochemistry [53]. In our case, it was not necessary due to the location of the tumor in the oral cavity and given the fact that there was no communication with the sinonasal tract, the absence of osseous destruction, in addition to the patient not presenting corresponding symptoms.

The diagnosis of a metastatic lesion in the oral region is a challenge both in the recognition as a metastatic lesion and in determining the place of origin. Recent advances in imaging technologies, molecular profiling tools, and immunohistochemical tests improve the identification of the primary site of origin and impact treatment options [54]. In our patient, the immunohistochemical technique showed positivity for CK20, helping to identify the origin of the primary tumor, and which was later confirmed with a colon biopsy. Therefore, the definitive diagnosis must be made based on microscopic features correlated with clinical characteristics, image exams, and immunohistochemical analysis. Important markers for the diagnosis of metastatic tumors of gastrointestinal origin include cytokeratin (CK) 20, caudal-type homeobox transcription factor 2 (CDX2), and cytokeratin (CK) 7 [38]. The CK7-negative and CK20-positive phenotype is found in the vast majority of well-differentiated or moderately differentiated large intestinal adenocarcinomas [55]. CDX2 is a sensitive and specific marker for colorectal adenocarcinoma, but its expression may be decreased between high-grade and stage tumors [56].

The treatment for cases of oral metastasis is surgical resection, which can be combined with radiotherapy and/or chemotherapy. However, treatment is often palliative, contributing to the patient’s quality of life [3]. According to this literature review, the death rate was high, as demonstrated in our case report.

Oral adenocarcinoma metastases are rare but should be included in the differential diagnosis even when there is no history of a primary tumor, as they may be a sign of cancer recurrence or the first manifestation of an occult primary neoplasm.

## Data Availability

The datasets used and/or analyzed during the current study are available from the corresponding author upon reasonable request.
